# Increased optical pathlength through aqueous media for the infrared microanalysis of live cells

**DOI:** 10.1007/s00216-018-1188-2

**Published:** 2018-07-02

**Authors:** James Doherty, Zhe Zhang, Katia Wehbe, Gianfelice Cinque, Peter Gardner, Joanna Denbigh

**Affiliations:** 1 Manchester Institute of Biotechnology, University of Manchester, 131 Princess Street, Manchester, M1 7DN UK; 2 School of Chemical Engineering and Analytical Science, University of Manchester, Oxford Road, Manchester, M13 9PL UK; 3 Diamond Light Source, Diamond House, Harwell Science and Innovation Campus, Didcot, Oxfordshire, OX11 0DE UK; 4 Biomedical Research Centre, School of Environment and Life Sciences, University of Salford, Salford, M5 4WT UK

**Keywords:** Synchrotron radiation (SR), Fourier transform infrared spectroscopy (FTIR), Infrared microspectroscopy (IRMS), Cancer, Single cell, Drug-cell interactions

## Abstract

The study of live cells using Fourier transform infrared spectroscopy (FTIR) and FTIR microspectroscopy (FT-IRMS) intrinsically yields more information about cell metabolism than comparable experiments using dried or chemically fixed samples. There are, however, a number of barriers to obtaining high-quality vibrational spectra of live cells, including correction for the significant contributions of water bands to the spectra, and the physical stresses placed upon cells by compression in short pathlength sample holders. In this study, we present a water correction method that is able to result in good-quality cell spectra from water layers of 10 and 12 μm and demonstrate that sufficient biological detail is retained to separate spectra of live cells based upon their exposure to different novel anti-cancer agents. The IR brilliance of a synchrotron radiation (SR) source overcomes the problem of the strong water absorption and provides cell spectra with good signal-to-noise ratio for further analysis. Supervised multivariate analysis (MVA) and investigation of average spectra have shown significant separation between control cells and cells treated with the DNA cross-linker PL63 on the basis of phosphate and DNA-related signatures. Meanwhile, the same control cells can be significantly distinguished from cells treated with the protein kinase inhibitor YA1 based on changes in the amide II region. Each of these separations can be linked directly to the known biochemical mode of action of each agent.

**Figure:**
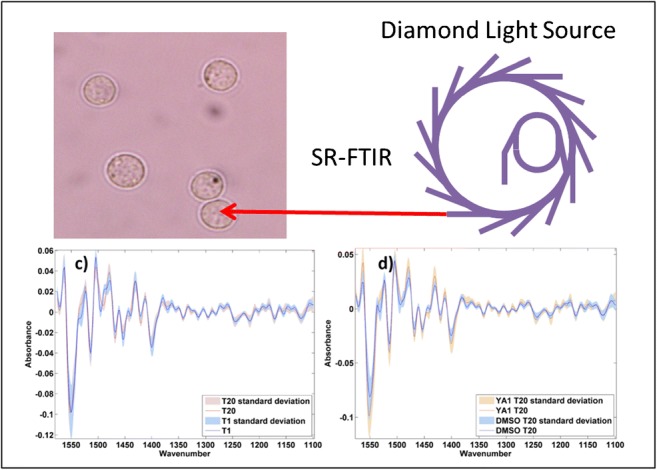
Graphical abstract

Graphical abstract

## Introduction

The use of Fourier transform infrared spectroscopy (FTIR) for the study of biological materials such as tissue, cells, plasma and serum is well established. Infrared (IR) spectra of biological materials have been used to obtain diagnostic and prognostic information on a range of diseases [1–7], as well as for the study of the effectiveness and mode of action of novel treatments [8–10]. Cancer has been a particular focus, with FTIR explored as a means to both improve diagnosis and inform the design of new treatments.

A significant body of work has demonstrated the ability of IR spectra to provide information on the mode of action of novel chemotherapy agents and assess their effectiveness against different cancer cells [11–13]. Additional work has also investigated drug-resistant cell lines and examined the effects of cell cycle on the uptake of certain drugs [14, 15].

Historically, the majority of cell studies using FTIR have relied on chemically fixed, dried samples. The benefits of this are clear; samples can be easily handled post fixation, and the same sample can be returned to multiple times for repeat measurement, given that IR is a non-destructive method of investigation.

However, chemical fixatives have been shown to have an effect on various structures within the cell, limiting the interpretation of resulting spectra [16–18]. Studies of sample dehydration also note changes in peak position, intensity and ratio across the spectrum [19–21]. Cell dehydration can particularly affect DNA bands, with the broader, weaker A-form DNA bands being more prevalent in dehydrated cell spectra, making DNA signatures harder to separate from other spectral contributions from proteins, RNA and carbohydrates [22, 23]. While the biochemical nature of the cell may be broadly maintained, subtle differences within a sample or as a result of stimuli may be lost. Studies of living cells have been able to yield biological and morphological details that were not accessible using fixed samples, particularly when combined with the brilliance of synchrotron radiation (SR) as a source [24–28].

The requirement of an aqueous environment to maintain cell viability is a significant constraint to FTIR analysis of live cells. This introduces the strong absorbance pattern of water into the spectrum in the ~ 1650 and 3000–3500 cm^−1^ wavenumber ranges, due to OH bending and stretching modes, respectively, which obscures much of the cell spectrum and makes extraction of biochemical information extremely difficult [29].

The water spectrum is a problem for analysts for two primary reasons: (1) the strength of the water absorptions causes insufficient light to penetrate to the sample, giving a signal that is too low to obtain quality data, and (2) the position of the water absorption signatures obscures key biological information relating to the amide and lipid bands arising from cellular species [30, 31].

Some work using living cells in aqueous environments has simply ignored the spectral regions most affected by water [32], but this is clearly severely limiting due to the significant amount of biochemical information being lost.

The removal of water from the acquired spectrum is a non-trivial issue. The subtraction of a pure water spectrum is not ideal, as the spectrum of separate bulk water will be different from that of water interacting with a biological system [33]. Likewise, removing the entire water contribution from the spectrum is also imperfect, as structural water accounts for approximately 70% of the mass of an average cell [34]. A number of water correction methods have been proposed, but with a lack of consensus over a single preferable method.

One method, published by Vaccari and colleagues [18, 35], removes a scaled water spectrum, in which the scaling factor is determined by an algorithm that optimises baseline flatness in the 1800–2500 cm^−1^ region, which contains no biochemical information but does contain the water combination band. The region containing the water combination band can, however, be heavily influenced by baseline variations.

Quaroni et al. were able to track a range of cellular metabolites by taking a reference spectrum through a cell-free area of growth medium, removing a fraction of the water contribution by a ratio to the background taken through an empty sample holder and then analysing the IR images in second derivative in order to highlight small spectral changes [36]. However, this does not take into account the different quantities of water present across the field of view of several cells and is therefore prone to under-/over-correcting for the bulk water contribution.

In separate work, Gelfand et al. [37] attempt to account for this over-correction by iteratively re-adding water contributions to their corrected spectra until the differences in baseline on either side of the C-H alkyl stretch at 2900 cm^−1^ was minimised. However, this work relied on a spacer of just 4 μm, which is a significant compression for the majority of cells, thereby likely to be picking up biochemical changes due to cellular stress and not mode of action of drug alone.

Deuterated water (D_2_O) has been proposed by some as a possible alternative medium to remove the water problem, due to its similar physical properties but significantly shifts absorption bands, allowing both a clear interpretation of the amide I band and a thicker bulk fluid layer of up to 20 μm [38]. With the exception of studies focused on isotopic exchange, such as deuterated protein or lipid content in D_2_O-resilient cells, the resulting red shift in the amide I band [39] and the overall toxic effects of culture viability and biochemistry over time [40–43] render D_2_O an unsuitable bulk fluid for drug-cell interaction studies.

In this study, we have tested an in-house water correction algorithm on spectra obtained from different thicknesses of bulk aqueous solution. There is little consensus on an optimum spacer size for IR analysis of living cells, with work published featuring spectra from spacers as small as 4 μm [37] and as large as 20 μm [36]. While the precise effects on cell viability and biochemistry will vary with the natural size of the cell and other factors, it is known that compression of the cell can have an effect on the resulting spectra [35]. This includes variations in the amide I/II peak height ratio and changes in protein and lipid concentrations, which, under severe deformation, can be permanent. Therefore, reducing the compression of the cell as much as possible, without sacrificing spectral quality, is important to maintaining the cell in as close to a natural environment as possible.

The problem of strong water absorption can be overcome through the use of a synchrotron source. The improved quality of spectra, in terms of both spatial and spectral resolution [44] and signal-to-noise ratio [26, 27], is well known, while the increased brightness generated by a synchrotron source relative to a thermal source [45] provides sufficient brilliance of light to penetrate a bulk water layer and still provide a cell spectrum with good signal-to-noise ratio.

Using an in-house water correction algorithm and working with the B22 Multimode Infrared Imaging and Microspectroscopy (MIRIAM) beamline at Diamond Light Source (DLS), we have tested our water correction procedure on hydrated, but chemically fixed, LNCaP prostate cancer cells in 6 and 12 μm spacers to evaluate the effect of pathlength on resulting biological spectra. Furthermore, we have studied the action of two novel drug treatments on live, hydrated K562 acute myeloid leukaemia (AML) cells with a pathlength that reduces compression of the living cells during analysis.

By using fixed LNCaP cells, in a liquid sample holder, we reduce the potential for biochemical variation between sample replicates, and therefore, differences in spectra should be the result of differences in sample holder loading alone. This gives an indicator of the reproducibility of our method over multiple experiments/replicates.

Meanwhile, poor response rates to conventional chemotherapy and low overall survival rates [46] make AML a focus for new and redeployed therapies [47], especially due to the high toxicity of conventional chemotherapy to older, frailer patients, who make up a significant proportion of sufferers [48].

Colleagues at the University of Salford provided two novel anti-cancer compounds for evaluation of drug-induced biochemical changes at the cellular level: PL63, which is a DNA cross-linking agent and analogue of the commercial anti-cancer drug busulfan [49], and YA1, which is a protein kinase inhibitor [50]. Due to their significantly different modes of action, cells treated with each of these agents should be distinguishable from each other based on their IR spectra and therefore allows us to demonstrate a workflow fit for purpose for FTIR analysis of drug-induced changes at the cellular level in hydrated/living cells.

## Methodology

### Cell culture

#### Spacer evaluation

LNCaP prostate cancer cells were grown to approximately 90% confluency using RPMI 1640 medium, supplemented with 10 vol.% bovine serum, 1% l-glutamine, 1 mM sodium pyruvate and 10 mM HEPES solution, with 1 μg/ml of puromycin and 2.5 μg/ml of blasticidin antibiotics in T25 culture flasks. Flasks were incubated at 37 °C and 5% CO_2_. Cells were harvested using trypsin, washed three times with phosphate-buffered saline (PBS) and fixed in 4% formalin solution.

#### Novel drug study

K562 AML cells were grown in RPMI 1640 medium (+l-glutamine) with 10 vol.% bovine serum and 1 vol.% penicillin-streptomycin at 37 °C and 5% CO_2_ in T25 cell culture flasks.

### Drug treatment

#### Novel drug study

Twelve to twenty-four hours prior to drug treatment, cells were passaged to ensure they were in exponential growth when the drug was introduced. Flasks were treated with drug compounds as follows: PL63 was administered at a concentration of 21 nM, equivalent to the IC50 value of busulfan [51], as no reported IC50 specific to PL63 in K562 cells was available; YA1 was administered at the reported IC50 value of 6.2 μM [52]. Solutions of each drug compound were made up in dimethyl sulphoxide (DMSO) such that a dose of 1 μl/ml was added to the culture flask for both treatments; control flasks were administered the equivalent amount of DMSO.

### Preparation of cells

#### Spacer evaluation

Formalin-suspended LNCaP cells were centrifuged at 500 g to pellet the cells. The pellet was then washed three times with PBS to remove residual formalin, and the supernatant was discarded after the third wash, leaving the cell pellet in residual PBS. To ensure a suitable concentration of cells for analysis, the measured sample was taken directly from this residual.

#### Novel drug study

Drug-treated and control cells were removed from the incubator and harvested after 1, 10, and 20 h. The sample was centrifuged at 500 g to pellet cells, from which the growth medium was discarded. The pellet was then washed twice with PBS to remove residual medium and any residual extracellular drug. After the second wash, the supernatant was poured away to leave a cell pellet in a residual amount (~ 0.5 ml) of PBS, from which the measured sample was taken.

### Loading of liquid sample holder

#### Spacer evaluation

Samples were measured using 6 and 12 μm spacers in the modified liquid sample holder available on the B22 MIRIAM beamline at Diamond. In each case, an amount of sample slightly less than the calculated volume was pipetted onto a clean 25 mm diameter, 1 mm thick calcium fluoride (CaF_2_) window. Using less than the calculated volume prevents completely filling the available space, thereby ensuring a dry area is available for taking a background measurement once the sample holder has been assembled. The sample holder is assembled with o-rings (3 mm) at the base, the CaF_2_ slide onto which the spacer and sample are placed, followed by a second CaF_2_ slide of the same proportions, and a final o-ring (1 mm). This configuration ensures that pressure is evenly distributed across the holder.

#### Novel drug study

Samples were measured using a 10 μm spacer in the modified liquid sample holder available on the B22 MIRIAM beamline at Diamond. Cell sample (1.5 μl) was pipetted onto a clean CaF_22_ window of 25 mm diameter and 1 mm thickness. The sample holder was then assembled in the same configuration as described above.

### SR-FTIR measurements

For both studies, data were acquired in transmission using the × 36 objective/condenser optics on a Hyperion 3000 microscope coupled to a Bruker Vertex 80 FTIR spectrometer at the MIRIAM beamline B22 at DLS, using a liquid nitrogen (LN_2_)-cooled mercury-cadmium-telluride (MCT), high-sensitivity 50 μm pitch detector and 15 × 15 μm^2^ slit size at the sample.

Two hundred fifty-six co-added scans (circa 35 s) were used for both background and sample measurements. A dry area of the sample was used for the background scan. A minimum of 65 cells per sample loading were selected for measurement using OPUS 7 software, with a corresponding measurement of the bulk PBS layer taken from a cell-free area adjacent to each selected cell for use in water correction. This gave a minimum of 130 spectra in each measurement and equates to approximately 90 min measuring time for optimal spectral quality.

### FTIR data processing

#### Spacer evaluation

The spectral contribution of the bulk aqueous solution must be removed before any other processing or analysis of the data can be carried out. This is done with an in-house MATLAB-based water correction algorithm, which performs a least-squares fit of the 1500–1700 cm^−1^ range of the sample spectrum to a Matrigel reference. This is used to determine a coefficient by which the corresponding PBS spectrum is multiplied to give the ‘water to be removed (TBR)’, taking account of the different quantities of aqueous solution present in the cell and PBS spectra. The water TBR spectrum was then subtracted.

The water-corrected LNCaP spectra were then quality controlled, smoothed using a Savitzky-Golay filter with nine points of smoothing and vector normalised. Note that the close refractive index matching of the CaF_2_, water and cell means that reflection artefacts are minimised in the FTIR spectra and resonant Mie scattering from the cell is very much reduced compared with fixed, dried samples. As such, RMieS correction is not required [53–55].

#### Novel drug study

Following water correction, data were quality controlled, vector normalised, converted to the second derivative and then smoothed using a Savitzky-Golay filter, using a third-order polynomial with 13 smoothing points.

The effects of saturation of the water bending mode were observed in the amide I region of a number of spectra with thicker spacers. For simplicity, it was decided to remove this region from all of the spectra. The second derivative spectra were cut to the 1100–1575 and 2750–3000 cm^−1^ wavenumber regions; this removes the saturated water region, the region below 1100 which is noisy due to the low wavenumber cut-off resulting from the use of 2 mm equivalent CaF_2_ thick material, and the 1800–2800 cm^−1^ range which contains no biochemical information.

### Spectral analysis

#### Spacer evaluation

Mean spectra were computed for each loading in the 6 and 12 μm spacers. These were investigated for variations in peak height, position, shape and any noticeable baseline variations between each of the three replicate loadings using each spacer.

#### Novel drug study

After conversion to the second derivative, mean spectra were computed for each drug treatment and sampling time point. These were investigated for variations in protein, lipid and nucleic acid signatures, both over time (intra-sample) and between different treatments (inter-sample) at the same time point. The data were also analysed using canonical variate analysis (CVA) [56], retaining 21 principal components (PCs), equivalent to 95% of the variation in the lower wavenumber region of the second derivative spectra. Similar methodology, retaining 95% of the variance, has previously been employed for the classification of FTIR and Raman spectra [57, 58]. Fivefold cross-validation was applied to the spectra of control and drug-treated cells; this involves the dataset being split into five groups, with four used for training a classification model and the fifth then projected in as a test dataset. This is repeated five times, with each group acting as the test set once.

## Results and discussion

### Evaluation of spacer thickness for the analysis of single cells in an aqueous environment

**Fig. 1:**
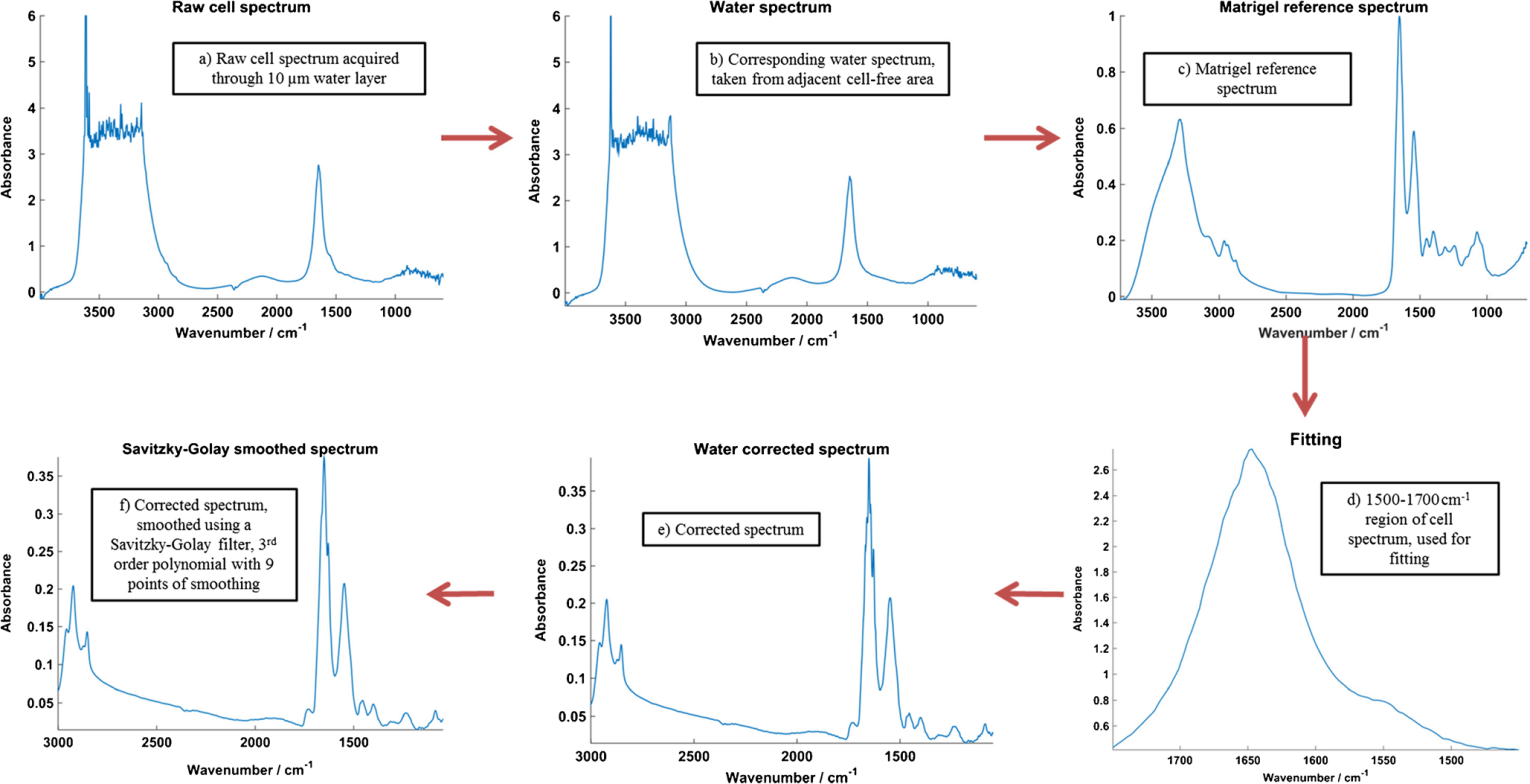
Schematic to show data processing workflow for the water correction algorithm

Development of our in-house water correction procedure involved testing on a range of cell types and experimental set-ups, as well as testing with a number of fitting ranges in order to optimise the procedure. Figure 1 shows a simplified schematic of the correction procedure using a single SR-FTIR example spectrum of a LNCaP cell.

**Fig. 2:**
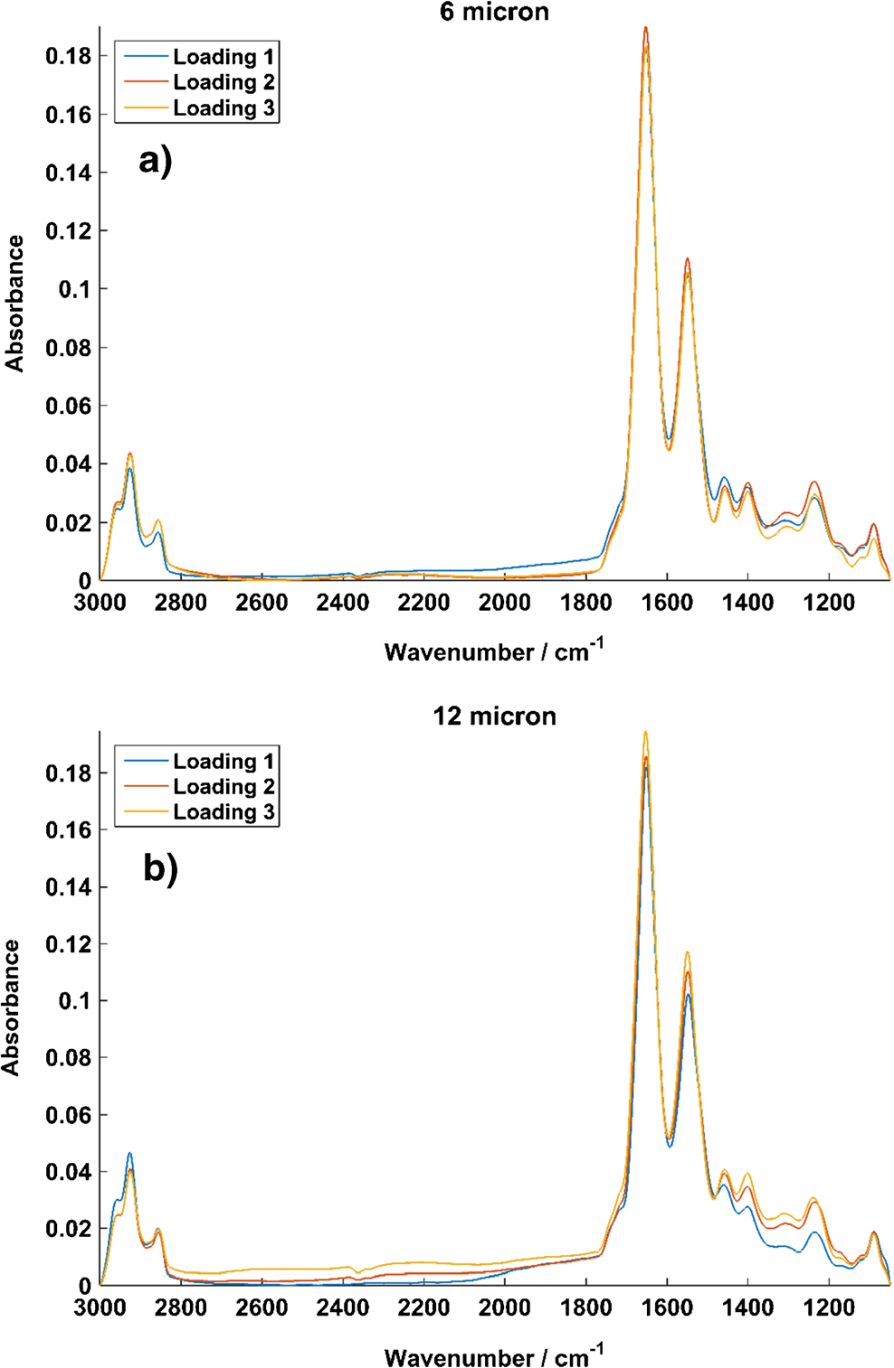
Vector-normalised mean spectra of 65 LNCaP cells from three repeat loadings of (**a**) a 6 μm spacer and (**b**) a 12 μm spacer. The 12 μm loadings show comparable quality and reproducibility, despite the significantly increased water contribution to be removed

The effectiveness of this in-house procedure on the spectra of formalin-fixed LNCaP cells, which were suspended in PBS and measured using both 6 and 12 μm spacers using SR-FTIR, is subsequently demonstrated. Figure 2 shows mean spectra for each loading of LNCaP cells using the 6 μm (Fig. 2a) and 12 μm (Fig. 2b) spacers. Firstly, this demonstrates the ability of our water correction procedure to extract good quality IR spectra from a water layer approaching the saturation limit, allowing us to reduce the compressive stresses on the cells by operating with a larger spacer.

The mean spectra from the 12 μm spacer show excellent reproducibility across the 2800–3000 cm^−1^ lipid region, consistent peak heights and positions across the amide I and II peaks and also show good consistency throughout the fingerprint region. There is some variation in the replicates in the lower wavenumbers of the fingerprint region, but the overall consistency and reproducibility of the spectra are consistent with the 6 μm repeat loadings, despite the significantly increased water contribution. Key cellular features have also been retained throughout the spectrum, despite measurements being taken at close to the water saturation limit.

### Observation of novel chemotherapeutic drug modes of action in hydrated cells at the single cell level

Having developed a water correction procedure and sample loading conditions that are able to consistently retain important spectral features, as demonstrated by Fig. 2, the next stage of our study is to test the algorithm on a live cell system. We selected two novel chemotherapy agents, PL63 and YA1, and monitored their effect on live K562 AML cells after 1 and 20 h of treatment.

Having determined through our analysis of spacer thicknesses that good-quality spectra could be obtained at a pathlength above 10 μm, the K562 cells were measured using a 10 μm spacer to reduce the risk of cell movement during measurement. This is due to the slight difference in the average size of LNCaP cells compared to K562; LNCaP cells have been observed to be up to 20 μm in diameter [59], while K562 cells are generally 15 μm in diameter [60].

**Fig. 3:**
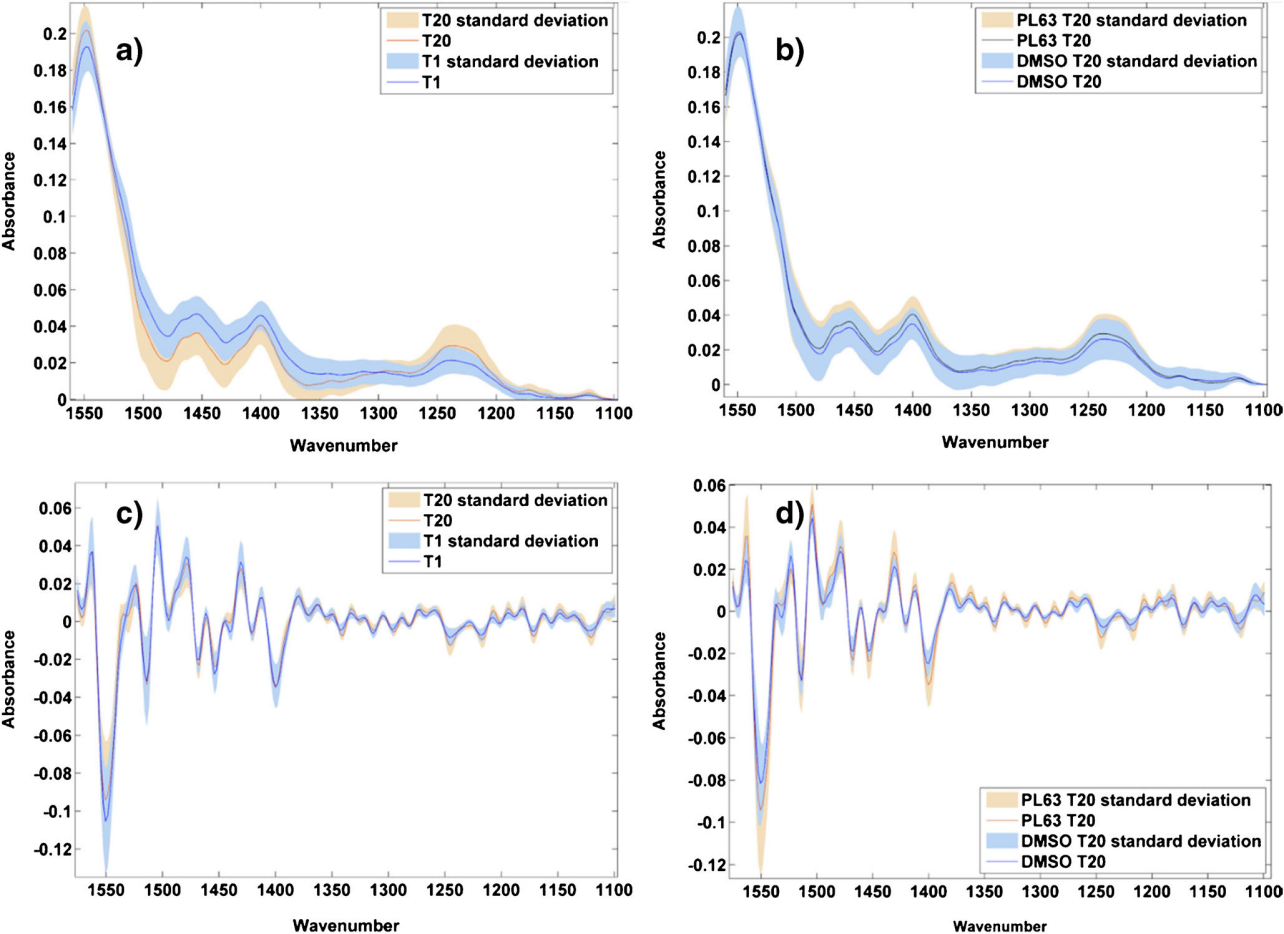
Normalised mean spectra of 120 cells, from three replicates, overlaid for PL63-treated cells after 1 and 20 h of drug treatment (**a**) and after 20 h of incubation with DMSO and drug (**b**). The corresponding second derivative spectra are shown in (**c**) and (**d**) to enhance spectral features corresponding to biological changes with drug treatment. The standard deviation of each mean spectrum is shown by the shaded area

Figure 3 shows normalised mean spectra of PL63-treated cells after 1 and 20 h, and DMSO control and drug-treated spectra after 20 h (Fig. 3a, b), along with the second derivative of each (Fig. 3c, d).

Examination of Fig. 3a, b shows no obvious regions of difference between the spectra, either over time or between the control and drug-treated spectra after 20 h. Conversion to the second derivative to enhance spectral features, however, removes any remaining baseline variations and highlights some more interesting biochemical changes. In Fig. 3c, the second derivative spectra are consistent across the majority of the range, apart from in the 1170–1250 cm^−1^ wavenumber range, where the signal from the 20 h spectrum is more intense than at 1 h. This region contains a number of features, including the amide III region and other proteins, but the two strongest signatures, at 1217 and 1244 cm^−1^, relate to phosphate stretching in DNA and RNA. This is of particular interest, as PL63 and busulfan—the commercial drug from which it is derived—are known DNA cross-linkers. These differences occurring in the mean spectra over time appear to be directly linked to the mode of action of the agent.

In Fig. 3d, the variations between the control and drug-treated mean spectra are more varied across the spectrum, with greater variation in some of the protein bands than in Fig. 3c, but the specific variations in the phosphate DNA/RNA peaks can still be seen, again linking these variations to the action of the drug.

**Fig. 4:**
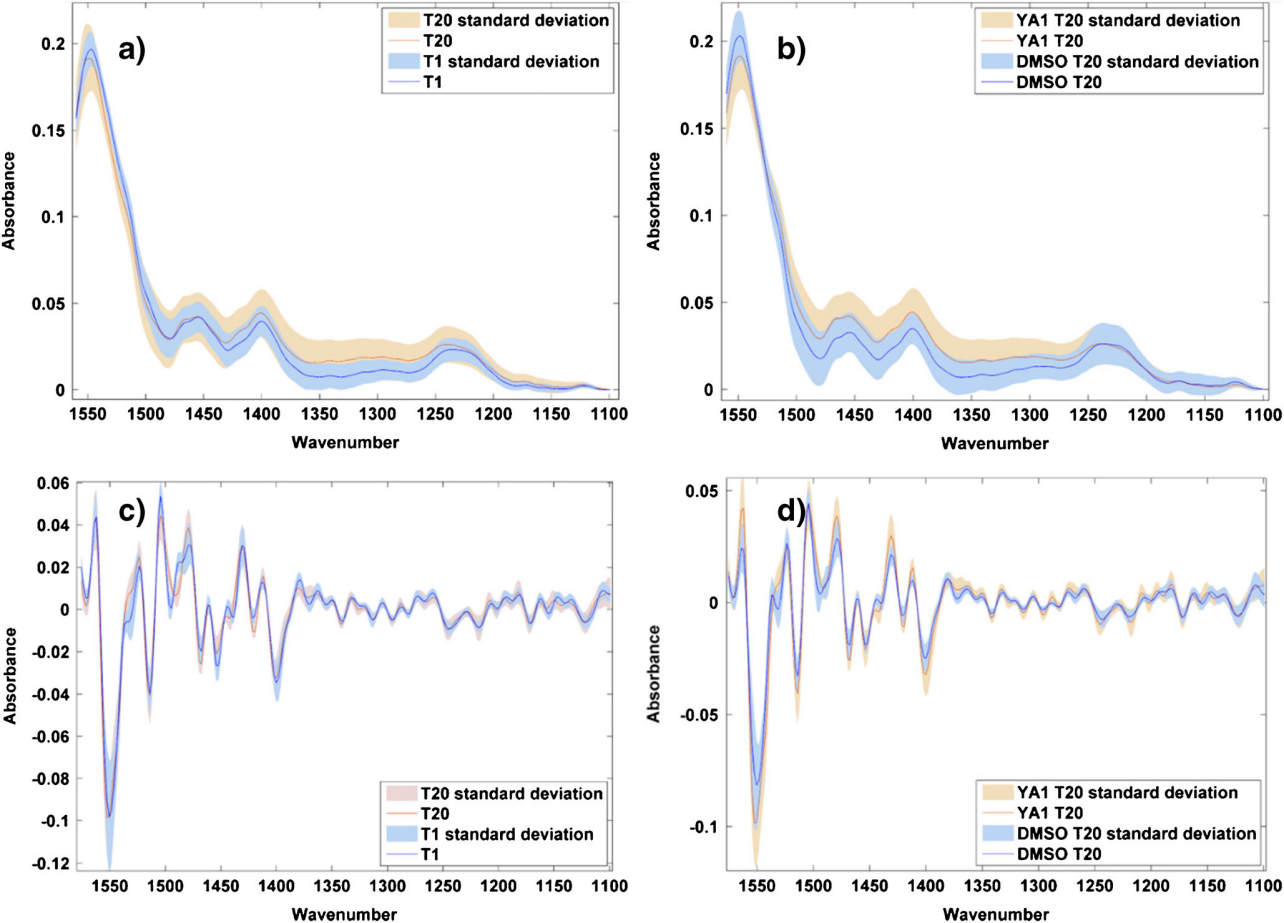
Normalised mean spectra of 120 cells, from three replicates, overlaid for YA1-treated cells after 1 and 20 h of drug treatment (**a**) and after 20 h of incubation with DMSO and drug (**b**). The corresponding second derivative spectra are shown in (**c**) and (**d**) to enhance spectral features corresponding to biological changed with drug treatment. The standard deviation of each spectrum is shown by the shaded area

Figure 4 shows the mean spectra of YA1-treated cells after 1 and 20 h (Fig. 4a), alongside the mean spectra of DMSO-treated control cells and YA1-treated cells after 20 h (Fig. 4b), with the corresponding second derivative spectra shown below (Fig. 4c, d).

The underivatised spectra do not show signs of obvious biochemical differences; the variations in the spectra in Fig. 4a, b could be attributed to baseline variations. However, in the second derivative spectra in Fig. 4c, d, differences again become more apparent. Significantly, the variations in the DNA/RNA bands noted in the PL63-treated cells are less pronounced, particularly in Fig. 4c when compared to Fig. 3c. The variations in Fig. 4c, d are less specific than those in the PL63-treated spectra but do include the amide II region at 1480–1560 cm^−1^. This is significant, given that YA1 is known to be a protein kinase inhibitor and would therefore be expected to induce changes in protein structure.

**Fig. 5:**
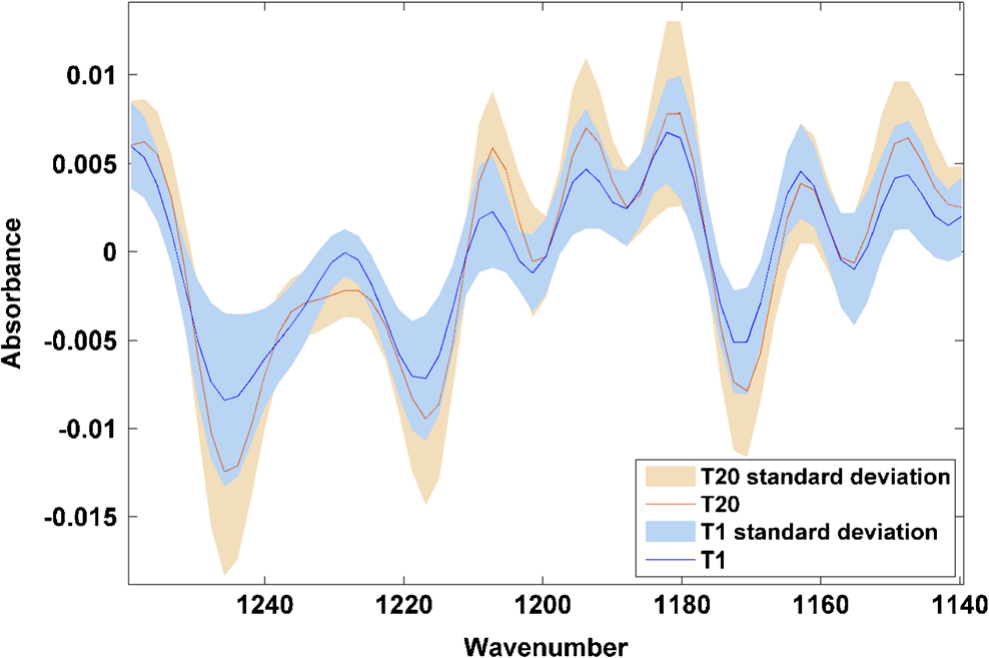
Enlarged region of mean spectra overlaid for PL63-treated cells after 1 and 20 h of drug treatment. Apparent drug-induced changes can be observed particularly at 1217 and 1244 cm^−1^ as well as from 1180 to 1210 cm^−1^

**Fig. 6:**
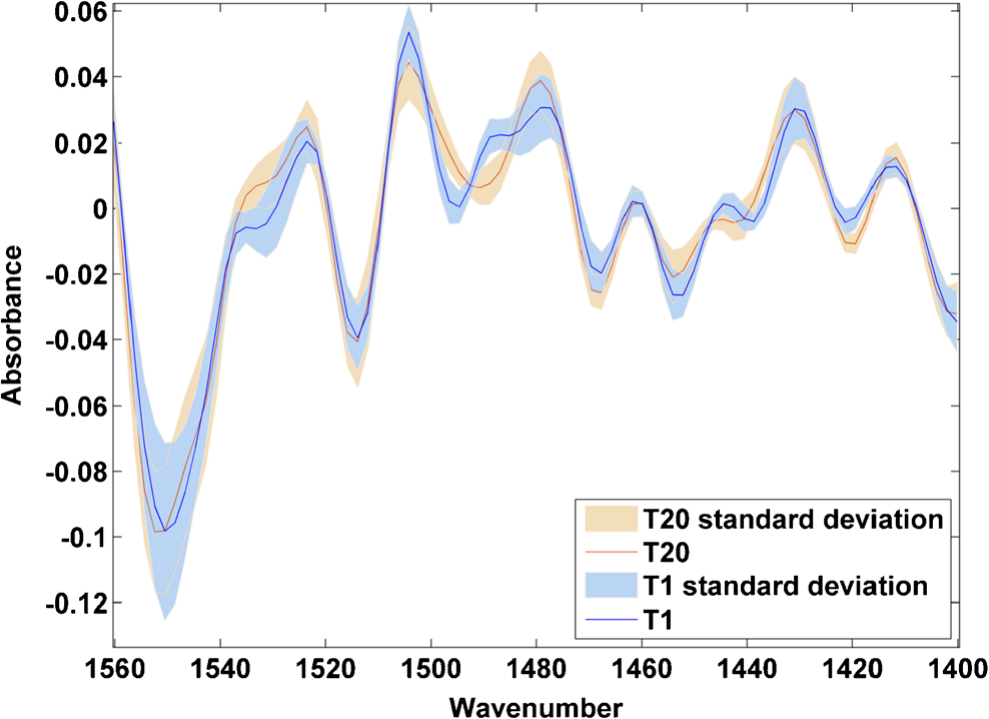
Enlarged region of mean spectra overlaid for YA1-treated cells after 1 and 20 h of drug treatment. Apparent drug-induced changes can be observed across the 1480–1560 cm^−1^ range, covering the amide II region

Figures 5 and 6 show the second derivative mean spectra of PL63- and YA1-treated cells, respectively, after 1 and 20 h of treatment, enlarged to the particular region of interest in each case—the phosphate and DNA/RNA bands in Fig. 5 and the amide II region in Fig. 6. The variations in mean spectra over time can be clearly seen in these enlarged images. Of particular interest is the variation in Fig. 5 of the spectra of PL63-treated cells at 1217 and 1244 cm^−1^, which corresponds with variations in DNA which would not be accessible if studying dehydrated cells.

**Fig. 7:**
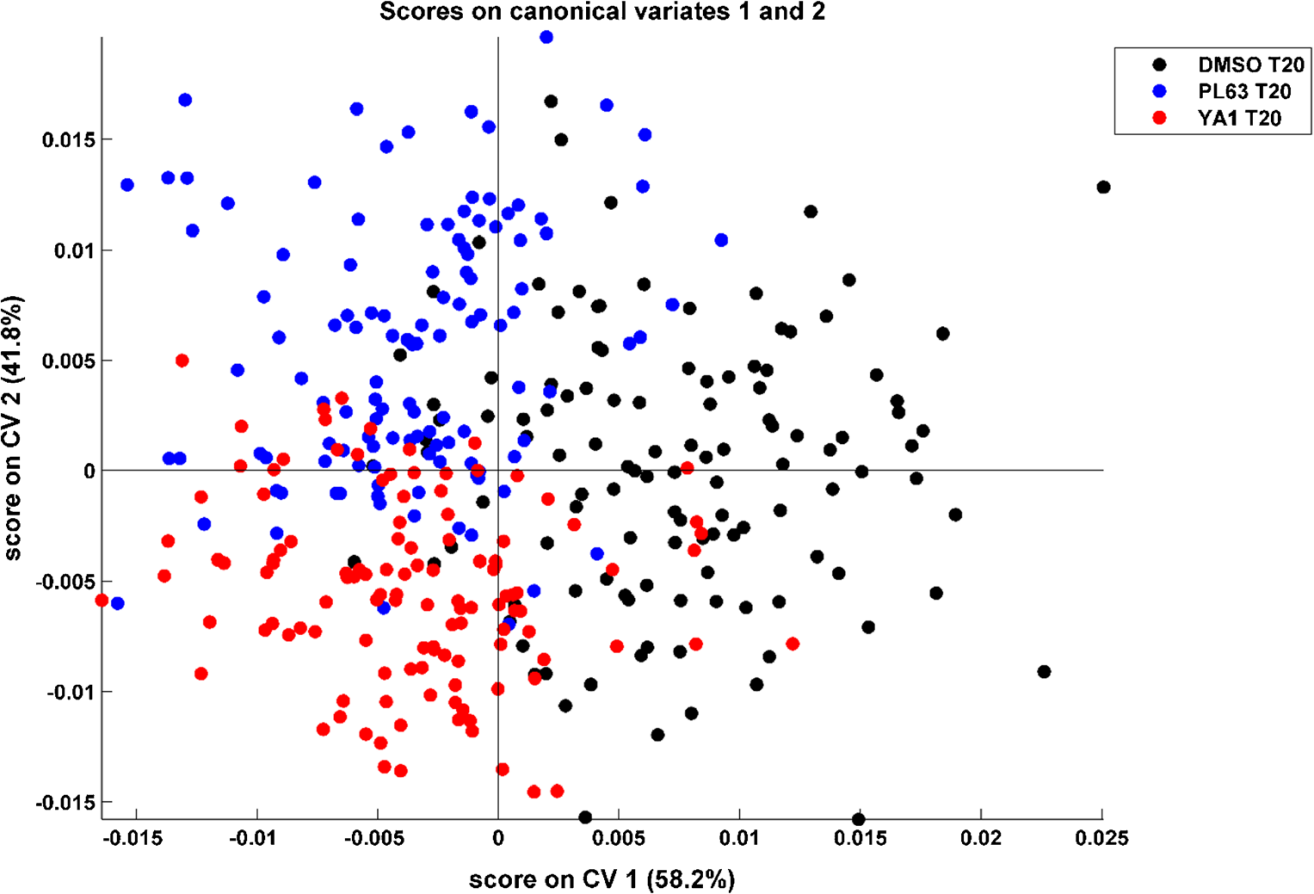
CVA score plot describing 95% of the variance of the second derivative data, showing grouping of DMSO-treated control cells (black) and cells treated with PL63 and YA1 (blue and red, respectively) after 20 h of incubation time

CVA was employed to assess differences in the response to the K562 cells to each drug. Figure 7 shows a CVA score plot, retaining 21 PCs, comparing spectra from control, PL63-treated cells, and YA1-treated cells after 20 h. Examination of the score plot shows discrimination between control and drug-treated cells across CV1, with grouping evident between the drug-treated classes across CV2. Spectra from 120 individual cells, from three combined replicates, were used in each class.

Multivariate analysis therefore demonstrates that the biochemical changes observed at the single cell level from each of the novel compounds tested can be classified in IR spectra relative to a control. Crucially, it is clearly demonstrated that that cells treated with each compound can be distinguished from each other. This is strongest indication that our live, hydrated cell system, with in-house water correction and using a 10 μm spacer, is able to differentiate between the modes of action of novel chemotherapy agents.

To further investigate the observed grouping in the CVA score plot, a fivefold cross-validation was performed on the data. The percentage of correctly classified spectra of each class, for each of the five folds, is shown in Table 1.

**Table 1:** Summary of percentage of test spectra correctly classified using *k*-fold cross-validation of second derivative spectra in the low wavenumber region, showing averages of 80% or greater correctly classified for each group

*k*	% correctly classified		
	DMSO T20	PL63 T20	YA1 T20
1	79	88	83
2	83	79	79
3	88	88	83
4	75	88	79
5	75	75	79
Range (%)	*75–88*	*75–88*	*79–83*
Mean (%)	*80*	*84*	*81*

Consistent with the score plot shown in Figure 7, fivefold cross-validation clearly shows the ability of SR-FTIR to distinguish between control and drug-treated cell spectra and also between cells treated with compounds with different modes of action. Fivefold cross-validation is able to correctly identify between 75 and 88% of control cells, 75–88% of PL63-treated cells, and 79–83% of YA1-treated cells. This is based on a relatively small sample size of 120 cells per class, giving five test sets of 24 spectra for each of the three classes, but clearly demonstrates clear spectral differences between control cells and cells undergoing different drug treatments. Given that all three sets of samples have been exposed to DMSO, some degree of similarity in the spectra is to be expected. By using effective water correction and then using the second derivative to highlight small variations in the spectra, we are able to observe spectral differences as a result of the exposure to different novel compounds. This is a promising development in our live cell methodology and demonstrates an ability to gain insight into the effectiveness and mode of action of novel compounds in living cells.

## Conclusions

This study has demonstrated an experimental protocol and water correction procedure that is able to obtain high-quality IR spectra from cells in a relatively thick aqueous layer, offering a significant improvement on many reported sample thicknesses for similar studies.

The key measure of any water correction is its ability to retain relatively subtle spectral changes between spectra. With our live cell study of K562 AML cells, we have been able to observe spectral changes in the second derivative between control and drug-treated cells, which can be directly related to the mode of action of that particular drug. Crucially, we have been able to observe differences in drug-treated spectra over time in spectral regions that would not have been observable if using dehydrated cells. For live cell analysis to continue to develop, a more in-depth level of analysis must be available when compared to fixed cells, to compensate for increased experimental complexity.

This experiment is the first employing our new protocol for hydrated cell studies using FTIR, developed in collaboration with the MIRIAM beamline at DLS. Future experiments will expand the range and type of cells investigated using the methodology.

Live cell analysis using SR-FTIR offers the ability to gain new insights into cell behaviour that cannot be obtained from fixed cells, despite the increased experimental complexity. Reducing the physical stress on cells is a significant step towards measurements in close to in vivo conditions as possible, thereby providing more reliable data for understanding drug-induced biochemical changes at a cellular level.

We have also been able to demonstrate the effectiveness of two novel anti-cancer agents on a particularly aggressive cancer type. This indicates, once again, the ability of FTIR to assess drug effectiveness and mode of action.
